# Decitabine impact on the endocytosis regulator RhoA, the folate carriers RFC1 and FOLR1, and the glucose transporter GLUT4 in human tumors

**DOI:** 10.1186/1868-7083-6-2

**Published:** 2014-01-09

**Authors:** David J Stewart, Maria I Nunez, Jaroslav Jelinek, David Hong, Sanjay Gupta, Jean-Pierre Issa, Ignacio I Wistuba, Razelle Kurzrock

**Affiliations:** 1 Head, Division of Medical Oncology, The Ottawa Hospital/University of Ottawa, 501 Smyth Road, Ottawa, ON K1H 8 L6, Canada; 2 University of Texas MD Anderson Cancer Center, 1515 Holcombe Blvd, Houston, TX 77030, USA; 3 Fels Institute for Cancer Research, Temple University, 3307 North Broad Street, Philadelphia, PA 19410, USA; 4 University of California San Diego Moores Cancer Center, 3855 Health Sciences Dr, La Jolla, CA 92093, USA

**Keywords:** Decitabine, RhoA, RFC1, FOLR1, GLUT4, LINE1 methylation, Promoter methylation

## Abstract

**Background:**

In 31 solid tumor patients treated with the demethylating agent decitabine, we performed tumor biopsies before and after the first cycle of decitabine and used immunohistochemistry (IHC) to assess whether decitabine increased expression of various membrane transporters. Resistance to chemotherapy may arise due to promoter methylation/downregulation of expression of transporters required for drug uptake, and decitabine can reverse resistance *in vitro*. The endocytosis regulator RhoA, the folate carriers FOLR1 and RFC1, and the glucose transporter GLUT4 were assessed.

**Results:**

Pre-decitabine RhoA was higher in patients who had received their last therapy >3 months previously than in patients with more recent prior therapy (*P* = 0.02), and varied inversely with global DNA methylation as assessed by LINE1 methylation (r = −0.58, *P* = 0.006). Tumor RhoA scores increased with decitabine (*P* = 0.03), and RFC1 also increased in patients with pre-decitabine scores ≤150 (*P* = 0.004). Change in LINE1 methylation with decitabine did not correlate significantly with change in IHC scores for any transporter assessed. We also assessed methylation of the RFC1 gene (alias *SLC19A1*). *SLC19A1* methylation correlated with tumor LINE1 methylation (r = 0.45, *P* = 0.02). There was a small (statistically insignificant) decrease in *SLC19A1* methylation with decitabine, and there was a trend towards change in *SLC19A1* methylation with decitabine correlating with change in LINE1 methylation (r = 0.47, *P* <0.15). While *SLC19A1* methylation did not correlate with RFC1 scores, there was a trend towards an inverse correlation between change in *SLC19A1* methylation and change in RFC1 expression (r = −0.45, *P* = 0.19).

**Conclusions:**

In conclusion, after decitabine administration, there was increased expression of some (but not other) transporters that may play a role in chemotherapy uptake. Larger patient numbers will be needed to define the extent to which this increased expression is associated with changes in DNA methylation.

## Background

Resistance to chemotherapy can arise from overexpression of resistance factors or from underexpression of factors required for drug efficacy [1,2]. Dose–response curve flattening at higher chemotherapy doses suggests that incurability of epithelial malignancies may be due primarily to underexpression of factors required for cytotoxicity [3]. If resistance were instead due to overexpression of resistance factors, one would expect a shoulder on a log-linear dose–response curve (with increasing efficacy at higher doses) instead of curve flattening at higher doses [1]. Examples of factors required for cytotoxicity that may be deficient in resistant cells include drug uptake transporters (for example, CTR1 for platinums and folate transporters for pemetrexed), drug activating enzymes (for example, deoxycytidine kinase for gemcitabine), obligate drug targets (for example, topoisomerase II for etoposide and doxorubicin) and pro-apoptotic molecules [2,4,5].

Deficiency of factors required for drug uptake and activation might directly potentiate resistance by reducing the amount of active drug in a cell, but could also have a secondary effect, in that these transporters may also play a role in uptake and cellular metabolism of nutrients. Hence, deficiency in these factors could reduce the rate of tumor cell division, and quiescent cells are generally more resistant to chemotherapy than are actively dividing cells [1,2,4]. Decreased uptake of several agents [6] and downregulation of expression of various transporters including folate binding protein and the endocytosis regulator RhoA [7] has been described in cisplatin-resistant cells that also have a reduced cell growth rate.

The mechanism by which transporters and other factors required for drug efficacy may be downregulated in cancer cells remains unclear. However, promoter hypermethylation is one mechanism by which gene expression may be downregulated, and hence DNA methylation could play a role in underexpression of factors required for drug efficacy [8-18]. Cancer cells often have abnormal hypermethylation and silencing of tumor suppressor genes [8-11] and of genes that support chemotherapy cytotoxicity [12,13]. Several genes may be hypermethylated in resistant cell lines [14,15] or tumors [18].

If DNA hypermethylation might play a role in resistance, then it follows that agents that reduce DNA methylation might sensitize cells to chemotherapy. The DNA demethylating agent decitabine reversed folate binding protein downregulation in cisplatin-resistant cells [7], augmented cellular uptake of methotrexate and carboplatin [7], and reversed resistance to various chemotherapy [15-17,19-24] or targeted agents [25] by upregulating expression of proapoptotic factors [19,20,25] and by other mechanisms [13]. DNA methylation also protected the anti-apoptotic factor survivin from repression by p53, and decitabine reversed this, permitting survivin repression by p53 [26]. Decitabine also partially reversed resistance to carboplatin in patients with advanced ovarian cancer [18,27,28].

The related DNA demethylating agent 5-azacytidine downregulated telomerase expression [29] and potentiated cisplatin [30-32], carboplatin [33], and docetaxel [31,32,34] preclinically by decreasing expression of pAKT [30,31] and other anti-apoptotic factors [31], by increasing expression of the tumor suppressor gene *TMS1*[34] and various proapoptotic factors [31,33], and by other mechanisms [30]. Also, 5-Azacytidine potentiated irinotecan in *p53*-mutant cells by upregulating expression of its obligate target topoisomerase-I via mechanisms involving p16 demethylation and Sp1 upregulation [35].

Decitabine is active clinically in myelodysplasia and leukemia [36-38]. Low-dose administration days 1 to 5 +/− days 8 to 12 of a cycle may be most effective [36-39]. Low-dose regimens are also particularly likely to induce DNA demethylation [38,39].

We administered low-dose decitabine days 1 to 5 +/− days 8 to 12 each cycle to patients with refractory malignancies and biopsied tumors before day 1 and on day 12 of cycle 1 [40]. In that study, decitabine decreased methylation of the long interspersed nuclear element 1 (LINE1) repetitive element (which was used as a surrogate for ‘global’ tumor DNA methylation) and increased tumor expression of the copper/platinum transporter CTR1. Pre-decitabine tumor expression of CTR1 was lower and LINE1 methylation tended to be higher in patients who were ≤3 months versus >3 months beyond most recent prior therapy [40]. Decitabine’s impact on CTR1 was greatest in patients more recently exposed to other agents. CTR1 expression correlated inversely with LINE1 methylation, although the CTR1 promoter was not methylated [40].

Based on our observations with CTR1 [40], we then asked whether other selected transporters behaved in a similar manner in these same tumor specimens. Specifically, we asked whether expression of other transporters is decreased in tumors of patients recently exposed to chemotherapy and targeted agents, whether tumor expression of transporters correlated inversely with LINE1 methylation, whether decitabine augmented transporter expression, and whether promoter methylation could explain any impact of decitabine on expression of one of these transporters.

## Results

### Patient characteristics

Patient characteristics have been reported in detail previously [40]. Tumor tissue was not available from all patients for all assessments, and hence patient numbers varied across assessments. Patient numbers varied slightly between different transporters since insufficient biopsy material was available for some assessments. Of the 31 patients treated on our decitabine trial [40], 27 had adequate tissue for at least one pre- or post-decitabine IHC assessment of one or more transporters. The 27 evaluable patients included 15 males and 12 females. Tumor types included cancers of the breast (four patients), kidney (three), head and neck (four, including two adenocystic carcinomas), lung (one), stomach (one), endometrium (one), and appendix (one), malignant melanomas (four), thymic neoplasms (three), neuroendocrine tumors (two), lymphomas (two), and desmoplastic tumor (one). Patients had received a median (range) of 5 (1 to 14) prior systemic regimens and a median (range) of 2 (0 to 6) prior targeted agents. Time from last prior treatment until entry unto this study varied substantially between patients. In patients with longer time intervals, this was primarily a function of relatively prolonged control or indolent tumor growth after discontinuation of their most recent prior therapy.

### Transporter immunohistochemistry scores and tumor type

Compared to other tumor types, adenocarcinomas had higher pre-decitabine IHC scores for reduced folate carrier (RFC1) (median score 240 in adenocarcinomas versus 80 in others, *P* = 0.0096). Adenocarcinomas also had a higher median pre-decitabine score for folate receptor-alpha (FOLR1) (90 versus 60, *P* = 0.0146). There were no major differences between adenocarcinomas and other tumor types for pre-decitabine scores for the endocytosis regulator/small GTPase RhoA (median scores 50 for both adenocarcinomas and others, *P* = 0.7758) or for the glucose transporter GLUT4 (median score 22.5 for adenocarcinomas versus 10 for others, *P* = 0.52).

### Transporter immunohistochemistry scores and time from last therapy

**Figure 1:**
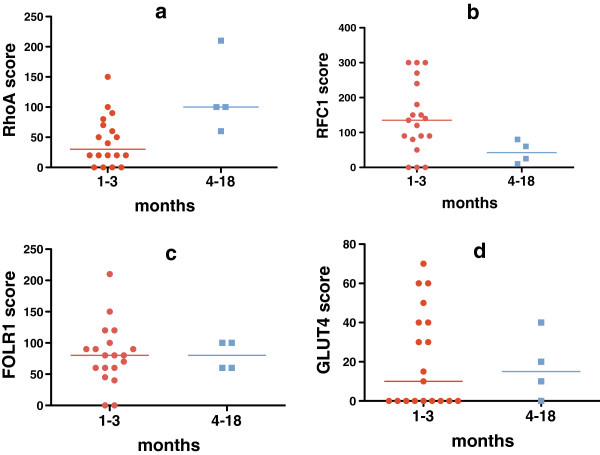
**Association of time from last prior treatment to tumor biopsy with pre-decitabine immunohistochemistry (IHC) scores.** Tumor expression (by IHC) for the small GTPase endocytosis regulator RhoA was higher with longer time intervals between prior therapy and biopsy (**a**, n = 18, median IHC score = 100 for patients with last prior therapy 4 to 18 months earlier versus 30 for patients with last prior therapy 1 to 3 months earlier, *P* = 0.02). Time from last prior therapy until pre-decitabine biopsy did not have a significant impact on tumor expression of the other transporters. For the folate transporter RFC1 (**b**, n = 19), the median score was 42.5 for patients with 4 to 18 months between prior therapy and biopsy versus 135 for those with a 1 to 3 month interval (*P* = 0.06). For the folate transporter FOLR1 (**c**, n = 19), median scores were 80 for both groups (*P* = 0.90). For the glucose transporter GLUT4 (**d**, n = 19), the median score was 15 for patients with 4 to 18 months between prior therapy and biopsy versus 10 for those with a 1 to 3 month interval (*P* = 0.97).

Based on our previous observation that pre-decitabine tumor IHC scores for CTR1 were significantly lower in patients who were ≤3 months versus >3 months beyond most recent prior chemotherapy or targeted therapy [40], we assessed whether pre-decitabine tumor IHC scores for other transporters were also higher in patients >3 months beyond last prior therapy. Results varied with the transporter assessed (Figure 1). Tumor scores for RhoA were higher in patients who had received their last therapy >3 months versus ≤3 months previously (median score 100 versus 30, *P* = 0.02), and correlated better with time from last therapy if both targeted therapy and chemotherapy were included (r = 0.24) than if only last chemotherapy was included (r = 0.16). Conversely, there was a trend towards tumor RFC1 scores being lower in patients with last prior therapy >3 months versus ≤3 months earlier (42.5 versus 135, *P* = 0.06). Scores did not vary with time from last therapy for FOLR1 (median scores 80 versus 80, *P* = 0.90) or GLUT 4 (median scores 10 versus 15, *P* = 0.97).

### Transporter immunohistochemistry scores versus LINE1 methylation (as a surrogate for global DNA methylation)

Based on our previous observation that tumor IHC scores for CTR1 correlated inversely with LINE1 methylation [40], we assessed whether pre-decitabine tumor IHC scores for other transporters also correlated with percent LINE1 methylation. Results again varied between transporters (Table 1). Pre-decitabine RhoA scores correlated inversely with LINE1 methylation, while there was no correlation with LINE1 methylation for the other transporters.

**Table 1:** Correlation of pre-decitabine immunohistochemistry (IHC) scores for transporters with percent LINE1 methylation

**Transporter**	**n**	**Spearman r**	***P***
RhoA	21	−0.58	0.006
RFC1	22	0.004	0.99
FOLR1	22	−0.15	0.50
GLUT4	22	−0.009	0.70

### Decitabine effect on transporter immunohistochemistry scores

**Figure 2:**
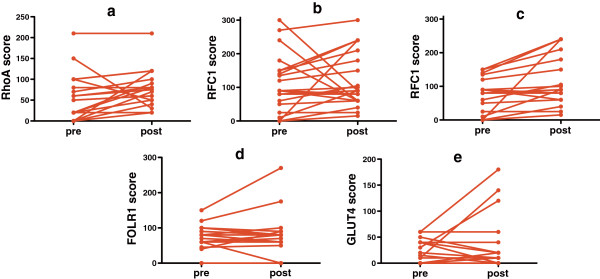
**Immunohistochemistry (IHC) scores for RhoA were significantly higher in post-decitabine tumor biopsies compared to pre-decitabine biopsies.** (**a**, n = 19, median scores 77.5 post-decitabine versus 50 pre-decitabine, with an increase in scores in 14 of 18 patients, *P* = 0.03). There was a trend towards higher scores in post-decitabine samples compared to pre-decitabine samples for RFC1. (**b**, n = 21, median scores 90 versus 90, with an increase in 14 of 21, *P* = 0.17). In patients with pre-decitabine scores ≤150, there was a significant increase in RFC1 scores with decitabine (**c**, n = 17, median scores 95 post-decitabine versus 80 pre-decitabine, with an increase in RFC1 scores in 13 of 17 patients, *P* = 0.004). There was no increase with decitabine in scores for FOLR1 (**d**, n = 19, median scores 80 in both pre- and post-decitabine samples, *P* = 0.89) or for GLUT4 (**e**, n = 20, median scores 10 post-decitabine versus 5 pre-decitabine, *P* = 0.61).

Based on our previous observation that tumor IHC scores for CTR1 increased after treatment with decitabine [40], we assessed whether tumor IHC scores for other transporters also increased post-decitabine (Figure 2). Following decitabine therapy, RhoA increased in 14 of 18 evaluable patients, from a median score of 50 to a median score of 77.5 (a relative increase of 55%, *P* = 0.03 by Wilcoxon signed rank tests). The median increase was slightly greater for patients whose last prior therapy was ≤3 months earlier than for patients whose last prior therapy was >3 months before decitabine initiation (median change 25 versus 10, *P* = 0.17).

RFC1 scores increased in 14 of 21 evaluable patients with decitabine therapy, although the median score remained unchanged at 90 (*P* = 0.17). However, if only patients with low pre-decitabine scores (<150) were assessed, then RFC1 scores increased modestly in 13 of 17 patients (from a median score of 80 to a median score of 90, *P* = 0.004). FOLR1 and GLUT4 scores did not vary significantly with decitabine therapy. Change in IHC scores did not vary significantly with decitabine dose (data not shown).

Impact of decitabine on transporter IHC scores did not appear to vary with tumor type. The changes in median IHC scores with decitabine were 30 (adenocarcinomas) versus 20 (others) for RhoA (*P* = 0.63), 30 (adenocarcinomas) versus 12 (others) for RFC1, -5 (adenocarcinomas) versus 0 (others) for FOLR1 (*P* = 0.89) and 0 (adenocarcinomas) versus 0 (others) for GLUT4 (*P* = 0.96).

### Post-decitabine changes in transporter immunohistochemistry scores versus changes in LINE1 methylation

In our earlier studies in this patient group, changes in tumor IHC scores for CTR1 did not correlate with change in LINE1 methylation [40]. Similarly, changes in IHC scores for other transporters did not correlate with change in tumor LINE1 methylation for any transporter assessed (Table 2).

**Table 2:** Correlation of change in transporter immunohistochemistry (IHC) scores with change in percent LINE1 methylation

**Transporter**	**n**	**Spearman r**	***P***
RhoA	20	−0.18	0.61
RFC1	19	0.22	0.37
FOLR1	17	−0.095	0.71
GLUT4	18	−0.18	0.48

### Decitabine effect on promoter methylation

Since IHC scores for RFC1 increased over the course of decitabine therapy in patients who initially had low scores, we assessed promoter methylation for its gene (alias *SLC19A1*) in patients with sufficient DNA. The *SLC19A1* assay (designed at the edge of the CpG island − 700 base pairs from the transcription start site) showed median methylation of 64% (range, 21 to 83%) in pre-decitabine tumor samples, compared to 18% in patient blood samples, 10% in normal control adult blood samples, 9% in control umbilical cord blood samples, and 63% in leukemia cell lines. Median *SLC19A1* methylation was 57.5% in post-decitabine tumor samples (range, 19-83%) (*P* = 0.63 in Wilcoxon signed rank test paired comparisons for 10 patients with both pre- and post-decitabine evaluable tumor samples). Changes in *SLC19A1* methylation did not vary with decitabine dose (data not shown).

*SLC19A1* methylation correlated with LINE1 methylation in 26 evaluable pre- and post-decitabine tumor samples (r = 0.45, *P* = 0.02), and there was a trend towards change in *SLC19A1* methylation varying with change in tumor LINE1 methylation in patients (n = 11) for whom both pre- and post-decitabine tumor samples were evaluable for both genes (n = 11, r = 0.47, *P* = 0.1457). While RFC1 protein expression did not correlate with *SLC19A1* methylation across all evaluable tumor samples (n = 24, r = −0.009, *P* = 0.97), there was a trend towards change in RFC1 protein expression varying inversely with change in *SLC19A1* methylation for patients in who both pre- and post-decitabine tumor samples were evaluable (n = 10, r = −0.45, *P* = 0.1912).

## Discussion

In earlier studies, we found in patient tumor samples that IHC scores for the copper/platinum transporter CTR1 increased with increasing time from exposure to last prior therapy [40], and we interpreted this as possible evidence that prior therapy might induce cross-resistance to platinums by downregulating CTR1, although other explanations for our CTR1 observation are possible. (For example, long time interval from last prior therapy to decitabine might also have been due to indolence of the patient’s tumor, and it is possible that high CTR1 expression could be a marker of tumor indolence such that patients with high CTR1 tolerated longer time intervals off therapy. However, in our earlier paper we found that high CTR1 expression correlated with high mitotic count [40], making this explanation unlikely). In our earlier paper, we also noted that tumor CTR1 IHC scores correlated inversely with LINE1 methylation (a putative marker of global DNA methylation), and that tumor CTR1 IHC scores increased after therapy with the DNA demethylating agent decitabine [40]. Liang *et al*. demonstrated a pleiotropic reduction in membrane transporters in platinum-resistant tumor cell lines [6], and Shen *et al*. reported that decitabine upregulated expression of a folate transporter and increased carboplatin and methotrexate uptake in one of these cell lines [7]. Several investigators demonstrated that decitabine or the related DNA demethylating agent 5-azacytidine can reverse resistance to chemotherapy in tumor cell lines, or can induce changes that might reasonably be expected to reverse resistance [13,19,20,25,26,29-31,33-35].

Based on these different lines of evidence, we hypothesized that exposure to a wide range of chemotherapy drugs or targeted agents might generate broad cross-resistance by downregulating expression of a range of unrelated membrane transporters. We further hypothesized that this transporter downregulation could occur through promoter hypermethylation and might be reversible by demethylating agents. Our findings from the current study failed to confirm our hypothesis. While our findings with the endocytosis regulator RhoA were very similar to our previous observations with CTR1, the other transporters behaved differently. Like CTR1 [40], RhoA scores varied inversely with LINE1 methylation and increased with time from last therapy exposure and with decitabine treatment, and others have reported that RhoA gene hypermethylation was associated with reduced RhoA expression in human tumors [41]. However, unlike CTR1 [40] and RhoA, scores for the transporters RFC1, FOLR1 and GLUT4 did not increase with time from last drug exposure and did not vary inversely with LINE1 methylation, although RFC1 did increase modestly with decitabine. Hence, exposure to a wide range of agents could potentially lead to a limited spectrum of cross-resistance by downregulating expression of some specific transporters, and decitabine could potentially increase expression of some transporters, but not others. It remains untested whether decitabine can reverse resistance to agents that rely on these transporters for uptake into tumor cells.

On the other hand, while a broad downregulation of transporters was not noted in our study patients with recent prior therapy exposure, we cannot comment on transporter function, and there are other factors that could come into play in limiting drug uptake. Specifically, a mislocalization of membrane proteins due to defective plasma membrane protein recycling has been noted in cisplatin-resistant cell lines [6], and this in turn was linked to defective endocytosis and to down-regulation of small GTPases including RhoA [7]. Hence, down-regulation of the small GTPases might potentially be sufficient to decrease uptake of a range of agents, and restoring RhoA and related factors could possibly reverse this, even if there is no obvious impact on expression of transporters.

The mechanism by which decitabine increased CTR1 scores (in our previous study [40]) and RhoA and RFC1 (in the present study) is not clear. Decitabine can increase expression of specific genes through mechanisms that are both dependent on [9] and independent of [9,11] promoter hypermethylation. We found no evidence of *CTR1* promoter hypermethylation in our previous study [40]. In the current study, we did find promoter methylation of the RFC1 gene (alias *SLC19A1*), but there was only a modest reduction in *SLC19A1* promoter methylation with decitabine. Furthermore, RFC1 scores did not correlate with *SLC19A1* methylation, although there was a trend towards change in *SLC19A1* methylation correlating with change in RFC1 score.

The reduced folate carrier is the major uptake mediator of anticancer antifolates and silencing of the reduced folate carrier gene (through a mechanism that appeared to be independent of promoter methylation) was noted in multiple resistant tumor cell lines [42]. Exposure of cell lines to methotrexate downregulated expression of RFC1, and this was not prevented by 5-azacytidine [43]. Since folic acid insufficiency alters DNA methylation [44], since there is an inverse relationship between folate levels and DNA methylation in human tumors [45], and since folic acid supplementation appears to induce DNA hypomethylation [46,47] in some circumstances (possibly by decreasing production of S-adenosylmethionine, the methyl donor for DNA methyltransferase) [47], it would also be of interest to assess whether addition of folic acid augments the ability of decitabine to induce DNA hypomethylation and restore silenced gene function. If decitabine can increase uptake of folate into tumors by increasing RFC1 expression, then folic acid and decitabine could possibly potentiate each other’s effects.

Overall, our observations suggest that it would be reasonable to test decitabine clinically in combination with other agents (including antifolates and platinums) to determine if it can prevent or reverse resistance that arises due to reduced drug uptake, and the experience to date in platinum-resistant ovarian cancer is encouraging [18,27,28]. It might be particularly useful to test its ability to potentiate chemotherapy in patients with low baseline expression of RhoA, RFC1 and/or CTR1, in those with higher baseline LINE1 methylation, and/or in those with a shorter time interval since last prior therapy. As noted previously, there are also several other mechanisms by which demethylating agents may prevent or reverse resistance to a variety of agents [7,13,14,19-21,25,26,29-31,33-35].

However, addition of decitabine to other agents could also have adverse consequences. For example, while we previously reported that decitabine therapy was associated with increased apoptosis in human tumors, we also found that mitoses and Ki-67 expression tended to increase with decitabine administration in tumors in which they were initially low [40] (suggesting that decitabine possibly might stimulate proliferation of quiescent tumor cells). While this might increase sensitivity of quiescent tumors to chemotherapy, it could also lead to resistance through accelerated repopulation. Furthermore, others have demonstrated that decitabine may reduce tumor cell sensitivity to cisplatin by reversing promoter-methylation-induced downregulation of the resistance factor glutathione-S-transferase-pi [48] and demethylating agents also increased tumor cell expression of resistance-associated drug efflux pumps, including MDR1/p-glycoprotein [49,50]. While some studies have suggested that DNA demethylation may increase efficacy of topoisomerase-1 inhibitors [35], others suggested that decitabine-induced DNA hypomethylation reduced camptothecin’s ability to induce DNA strand breaks [51]. The related agent 5-azacytidine augmented expression of the DNA repair enzyme ERCC1 by reducing its promoter methylation, and thereby decreased tumor cell sensitivity to radiation [52]. Tumor cell expression of metallothioneins (which may be important in chemotherapy resistance [4]) was also reduced by promoter methylation and augmented by 5-azacytidine [53].

## Conclusions

In summary, decitabine administration was associated with biological changes in human tumors that could prove therapeutically useful, particularly if decitabine is combined with other agents. However, as we have argued previously for anticancer therapies in general [54], the broad range of decitabine’s potential effects mean that in future studies we should use extensive molecular characterization of patients’ tumors to carefully assess which patients are most likely to be helped versus harmed by decitabine use.

## Methods

### Patients and methods

As previously reported [40], eligibility criteria for this study (approved by the University of Texas MD Anderson Cancer Center Research Ethics Board) included written, informed consent, diagnosis of incurable malignancy refractory to standard therapy, adequate organ function, and tumor amenable to biopsy. Decitabine (supplied under a Collaborative and Research Development Agreement by the National Cancer Institute Division of Cancer Treatment and Diagnosis) was administered intravenously over one hour [40]. Doses were 2.5, 5, or 10 mg/m^2^/day on days 1 to 5 and 8 to 12 each 4-week cycle or 15 or 20 mg/m^2^/day on days 1 to 5 each cycle. Filgrastim was added at higher doses.

Patients had tumor biopsies pre-decitabine and day 12, cycle 1 [40]. IHC was assessed using formalin-fixed, paraffin-embedded 5 μm-thick tissue sections that were deparaffinized and hydrated, then stained with mouse antibodies against RohA, RFC1, FOLR1 [55], and GLUT4 (Table 3). Envision Plus Dual Link-labeled polymer (Dako, Inc, Carpinteria, CA, USA) was used as the secondary antibody. Staining intensity was scored as 0 to 3+, then multiplied by the percent of tumor cells staining to give an IHC score of 0 to 300. Changes in IHC scores were calculated by subtracting the day 1 score from the day 12 score.

**Table 3:** Antibodies used for immunohistochemistry

**Protein**	**Antibody type**	**Source**	**Dilution**
RFC1/SLC19A1	Polyclonal (antibody 62302)	Abcam, Cambridge, MA, USA	1:100
FOLR1	Monoclonal IgG, clone Mb343	Homemade, kindly supplied by Dr. Wilbur Franklin [55]	1:500
GLUT4	Polyclonal	Abcam, Cambridge, MA, USA	1:1000
RhoA	Monoclonal	Novus Biologicals, Littleton, CO, USA	1:250

Percentage of DNA CpG islands that were methylated was assessed by LINE1 assays as a surrogate for global DNA methylation as previously described [56] and reported [40]. Change in LINE1 methylation was calculated (day 12 value minus day 1 value divided by day 1 value). Promoter methylation for *SLC19A1* (the gene encoding RFC1) was assessed by bisulfite pyrosequencing [57], using primers described in Table 4.

**Table 4:** **Primers used for pyrosequencing assessments of *SLC19A1 *** **methylation**

**Step**	**Name**	**Sequence**
PCR1	RFC1F1	AGGGATAAGTATAGTTTGTTTTTGGGGAT
PCR1	RFC1R	AATAACCCAAACCCCCCTTCC
PCR1	RFC1RU	GGGACACCGCTGATCGTTTAATAACCCAAACCCCCCTTCC
PCR2	RFC1F2	GTGATTAGTAAGGGTTTGTATTAAGGAGTAAG
PCR2	BioUni	[Biotin]GGGACACCGCTGATCGTTTA
PSQ	RFC1S	TAGTTTTTATTTTAGTAGGGATAG

Non-parametric two-tailed statistics were calculated using GraphPad Prism 5.0 (Spearman tests for correlations, Wilcoxon signed rank tests for paired comparisons, and Mann–Whitney tests for comparisons of two groups). Low patient numbers limited statistical power.

## Competing interests

None of the authors have relevant competing interests to declare.

## Authors’ contributions

DJS designed and oversaw the overall study, analyzed the data and drafted the manuscript. MIN performed all immunohistochemistry on tumor samples. JJ assessed LINE1 methylation and *SLC19A1* methylation of tumor samples. DH contributed to patient recruitment. SG oversaw tumor biopsies. JPI oversaw LINE1 methylation and *SLC19A1* methylation studies. IIW oversaw tumor sample collection, storage, retrieval and immunohistochemistry. RK oversaw patient recruitment. All authors read and approved the final manuscript.
